# The Hospital. Nursing Section

**Published:** 1904-07-30

**Authors:** 


### The Hospital

**Nursing Section.**

Contributions for this Section of "The Hospital" should be addressed to the Editor, "The Hospital" Nursing Section, 28 & 29 Southampton Street, Strand, London, W.C.

No. 931.—Vol. XXXVI. *SATURDAY, JULY* 30, 1904.

### Notes on News from the Nursing World.

### FROM THE "COURT CIRCULAR."

Buckingham Palace, July 21.—The Queen this afternoon presented certificates to nurses who have joined the National Pension Fund since 1901. The King, their Royal Highnesses the Duke of Sparta, the Princess Victoria, Princess Mary of Wales, and the two younger children of their Royal Highnesses the Prince and Princess of Wales were present with her Majesty. The nurses, formed up in the garden of the palace, received the King and Queen with a salute by raising the right hand, after which they passed before their Majesties, when the Queen presented the certificates, the recipients being named by Sir Henry Burdett. The ladies and gentlemen in waiting were in attendance. Colonel W. Campbell, A.D.C., Colonel Bor, R.M.A., Captain Cottingham, R.M.A., Lieutenant Wright, R.M.A., and Mr. L. H. M. Dick (secretary) had the honour of being presented to their Majesties.

### THE PENSION FUND FÊTE.

The reception by the Queen at Buckingham Palace of the nurses of the Royal National Pension Fund is described in detail elsewhere. To Mr. Dick and to the staff under him belongs much of the credit that all the proceedings went off without a hitch, though this, of course, would have been impossible without the willing and loyal co-operation of the nurses themselves. The railway companies likewise merit a word of recognition for their liberality in issuing return tickets at single fares from stations outside the metropolitan area, and thus enabling many members of the Fund to be present at the ceremony who otherwise would not have been able to afford the expense of a long journey. The secretary has requested us to mention that nurses who were not able to attend the reception to receive their certificates can have them sent by post if they will forward their names, their policy numbers, and a stamped envelope to him at the offices of the Pension Fund, 28 Finsbury Pavement, E.C.

### THE ROYAL RED CROSS.

The King has been graciously pleased to confer the decoration of the Royal Red Cross upon Miss J. Bradbury, Miss C. Addison, Miss S. Ruiter, and Miss E. M. Early, in consideration of the services rendered by them in tending the sick and wounded in the Volunteer Hospital at Intombi, during the late war in South Africa.

### THE QUEEN AND THE BRITISH HOME FOR INCURABLES.

The Queen's visit to the British Home and Hospital for Incurables at Streatham the other day was a surprise visit, and it speaks well for the officials that her Majesty found everything in good order, and expressed her satisfaction with all that she saw. The Queen was received by the Chairman of the House Committee, the Secretary, the members of the Hospital Committee, and the hon. secretary of the Ladies' Committee, and was conducted over the hospital by Dr. Scamm, the medical officer, and Miss Macdonald, sister-in-charge. To Miss Macdonald, we understand, was largely due the fact that the arrangements for the Queen's reception and inspection were all that could be desired. Miss Carlile, the oldest patient in the home, had the honour of presenting a bouquet to her Majesty.

### THE PROCEDURE OF THE SELECT COMMITTEE.

On Tuesday the Select Committee on Registration sat for the last time prior to the prorogation of Parliament. It is quite possible that they may not meet again. A Dissolution before next Session would, of course, render it necessary to begin *de novo*. If, however, the Government do not intend to appeal to the constituencies until next year, the Committee will, doubtless, be re-appointed. In that case we hope that the chairman and members will refrain from showing that their sympathies lie in a particular direction. In their report, if ever they issue one, they can state their views as fully as they please, but during the inquiry their business should be strictly confined to asking questions in a manner which does not suggest a preference.

### A RULE OF STUDY AT KING'S COLLEGE HOSPITAL.

A reprint of the nursing regulations at King's College Hospital having been rendered necessary by the removal of the rule requiring attendance at chapel, a rule of study, which has been arranged to assist the nurses in their private reading, has been incorporated. The subjects which are expected of every nurse, while covering the same ground as formerly, are so arranged as to avoid waste of time and desultory study. They include, for the first year, elementary science, chemistry and physics, elementary physiology and elementary anatomy, the first subject occupying two months, and the other two the remaining nine. In the second year the first nine months are devoted to the general principles of medical diseases and of surgical diseases and accidents, and the next two months to repetition of study in physiology and anatomy. The first two months of the third year are given to the general principles of gynæcological diseases, the second two to the diseases and nursing of children, the third two to diseases of the eye, ear, nose, and throat; the fourth two to hygiene, and the last three months to the repetition of study in medical and surgical diseases. There is also instruction in cooking for the sick and convalescent. This class-study is, of course, in addition to the lectures by the medical staff, given twice a week from November to June.

### THE NURSING QUESTION AT THE HEALTH CONGRESS.

The Earl of Radnor, in his presidential address at the annual congress of the Royal Institute of Public Health, which was held this year at Folkestone, alluded to the nursing question. He said that it seemed to him, on account of the Midwives Act alone, of almost essential importance that the provision of nurses should be increased, and, if possible, by private exertion and by the assistance of the private purse. There was no doubt, Lord Radnor thought, that when these nurses were provided, and when every country district and every village had its nurse, it would be of incalculable benefit to the country itself, and ultimately to the population at large. The Countess of Radnor presided over the ladies' section, in which the State registration of nurses was advocated.

### PRELIMINARY TRAINING AT TORONTO.

The first course of preliminary instruction for nurses undertaken in Canada has been arranged by the Toronto General Hospital and the Toronto Technical School. In a circular issued by the authorities of the Toronto General Hospital it is stated that "intending applicants to the Training School for Nurses are notified that after this date preference will be given to candidates who hold a certificate of the preparatory course, provided they are otherwise eligible." The preparatory course referred to consists of two terms of three months each. The classes are held daily in the Technical School, and the various branches in which intending nurses receive instruction are anatomy, physiology, medical chemistry, hygiene, bacteriology, dietetics, cookery, household economics, English language, and vocal expression. The question of the success of preliminary training for nurses in Toronto has already been answered to a certain extent, for there are now six nurses in training at the Technical School, applicants for the class in the Toronto General Hospital, which enters into training there next fall.

### WESTMINSTER HOSPITAL AND THE PRIVATE STAFF.

We certainly think that the primary object of the Westminster Training School and Home for Nurses should be the supply of nurses for the hospital, and the secondary one the provision of private nurses. Therefore, the approval by the Chancery Division of the High Court of Justice of the scheme put forward by the governing body of the Westminster Hospital and settled by the Attorney-General, which provides for this, is a matter for congratulation. It may be hoped that the detailed provisions with regard to the election and position of the lady superintendent of the home and the mode of securing the pension fund for the nurses, which still remains to be approved by the Court, will be submitted without delay.

### DISOBEDIENT MIDWIVES.

At the last meeting of the Central Midwives Board, who have adjourned until September 29th, the case of a disobedient midwife at Brighton was discussed. It was stated that she caused the death of a patient, and that, in defiance of the injunctions of the Medical Officer of Health, she went to another case from one suffering with scarlet fever. Apparently, she was allowed to continue her work as if nothing had happened. The Board very properly agreed that it was the duty of the local authority to have suspended her and communicated the facts to them at the time. It is possible that the clause of the Act dealing with such cases is not familiar to the local authority, though the regulations of the Act are readily obtainable by all. However, the secretary of the Board has been instructed to quote them at length and to ask for an official report. It is obvious that midwives who do not obey instructions can do a great deal of harm; and it is therefore of vital importance that instances of disobedience should not be lightly treated. Otherwise, the Act itself will speedily be discredited.

### NURSES' HOME AT THE CANCER HOSPITAL.

Good progress has been made with the new home for the nurses of the Cancer Hospital in Fulham Road, and it is hoped that it will be finished and ready for occupation by the autumn. Sleeping and sitting-rooms are provided for twenty-five sisters and nurses. On the entrance-floor there are two sitting-rooms, a small pantry for tea-making, etc., and two or three bedrooms, the other two floors being devoted to bed and bath-rooms. There is a balcony on the side opposite to the hospital, with an alcove underneath large enough for deck-chairs. The house will be lighted by electric light, and the rooms will be heated by hot air. There will be fireplaces in the sitting-rooms. An ancient mulberry tree, the pride of the garden, has so far been preserved. When the home is finished the entire staff will be accommodated outside the hospital building, the night superintendent and nurses being already housed in a separate home in the grounds.

### PROGRESS OF MATERNITY NURSING IN HONG KONG.

The wife of the Officer administering the Government of Hong Kong, Mrs. F. H. May, opened the Alice Memorial Maternity Hospital last month. It is connected with the London Mission, and is affiliated to the Alice Memorial and Nethersole Hospitals, to the latter of which it stands adjacent. The staff accommodation includes matron's quarters, and 10 rooms for nurses. Dr. Sibree, the lady doctor attached to the hospital, hopes to commence work outside the institution on September 1st, and will be assisted by Miss Langdon, a thoroughly trained and experienced nurse. Mrs. May, in declaring the building open, expressed the pleasure it gave her to perform the ceremony, and said that she thought the new hospital would confer a great benefit upon the Chinese community, and exercise a powerful influence in the spread of greater and more enlightened methods of midwifery.

### A NURSE'S SUCCESSFUL CLAIM.

At the Brompton County Court last Friday, Mrs. E. Cherry, a maternity nurse, brought an action against Mrs. Allpress, of 133 Ashley Gardens. The plaintiff stated that on March 7th last she had an interview with the defendant, and discussed a proposal that she should attend the lady in her approaching confinement. Ultimately she was definitely engaged for six weeks at a remuneration of three guineas per week, as from a date to be named in the second week in May. On April 27th, however, she received a letter from the defendant determining the engagement, in consequence of having made other arrangements. She claimed six weeks' remuneration, and one guinea per week for six weeks in respect of board expenses. The defendant's case was that there was no engagement of the plaintiff's services, either at the interview on March 7th or subsequently. Judge Lloyd came to the conclusion that the plaintiff's recollection of the arrangement was correct, and he gave judgment in her favour, with costs. An attempt on the part of counsel for the defendant to obtain a deduction of the amount on the ground that the plaintiff was not, at any rate, entitled to six guineas for board expenses, was unsuccessful.

### "POUND DAY" AT A COTTAGE HOSPITAL.

The experience of the Matron of a Devonshire cottage hospital, which has just had a singularly successful "Pound Day," may be of service to other institutions in need of money. The day set apart was July 13th, and the time for receiving contributions from 2 to 6 p.m. The hospital swarmed with old patients and new friends, all bringing parcels, until the common room looked more like a small Army and Navy stores than a general sitting-room. The idea appealed to many who were willing and eager to help the hospital in which they or their friends had been treated, but who did not like to give their small sums of money. One burnt child brought no fewer than three pounds of coppers which she had begged, in addition to her mother presenting large stores of groceries. A girl who had been rescued from consumption brought her collection, over a sovereign's worth. There were several favourite gifts —viz., lump sugar, soap, tapioca, tea, butter, eggs; of those there were great quantities, and everything seemed represented except salt. The gifts numbered over 300, but the parcels were quite 500. There were two curious "pounds"—one of snuff and one of clergymen's ties—and many pounds of coals were also received. The coppers turned the scale at over 11 pounds, and the donations amounted to upward of £36. School children especially seemed to enjoy the collecting very much, and the device seems a useful way of helping a small hospital. The day following a sale of work was held, when all the most perishable things, such as fruit, vegetables, and about 20 of the 36 lb. of butter were sold, the extra provisions providing the teas. Even then the result of "Pound Day" was not finished; for there were the surplus stores to be attended to, the butter to be salted, the eggs to be preserved, the soaps to be put by in wooden boxes, the sugar in a cask, the tea in tin boxes, and all the rest in jars. The money raised was over £60, and the stores will last a long while.

### QUEEN'S NURSES AT HUDDERSFIELD.

Two remarkable facts in connection with the work of the six Queen's nurses in Huddersfield are that, as the Chairman pointed out at the annual meeting of the District Association, there were 109 operations during the year, and that there were 144 deaths out of 730 patients. These figures show the very serious nature of the cases which the nurses had to deal with. It may be mentioned that 649 were recommended by medical men. The nurses paid nearly 16,000 visits. As to the financial position of the association, the committee spent £30 more than they received during the year, but as they started with £40 in hand, there is no deficit. Amongst the donations received was one of five guineas from a gentleman as a thankoffering for his mother attaining the age of three score years and ten.

### NIGHT NURSING AT PENZANCE INFIRMARY.

At the last meeting of the Penzance Guardians it was reported that a nurse who had been engaged had declined to take up the appointment on the ground that she did not approve the cooking arrangements and the absence of assistance for night nursing. A member of the board, commenting on these objections, said that a nurse could not be expected to be up day and night, and that, "unless a patient howled sufficiently loud to awake the nurse from her sleep, he or she would be likely to go without nursing until the morning." The question was referred to the visiting committee, but the prospect of patients howling in the night for help is sufficient, apart from any possible defects of diet, to deter the average nurse from accepting an engagement at the Penzance Infirmary.

### HEREFORDSHIRE HOSPITAL.

The report of the Finance Committee of the Board of Management of the Herefordshire General Hospital, in respect to the private nursing staff, to which reference was made in our issue of July 2nd, came up for discussion at the July meeting of the board, and was eventually unanimously adopted. Incidentally it was stated that if it had not been for this scheme a nursing home would have been opened in Hereford before now. The opening of a nursing home is still a possibility; but it having been made perfectly clear that the hospital will not derive a profit from the private nursing fund, but only be recouped for money expended, we do not see why the arrangement decided upon should not work smoothly and to the advantage of the nurses, the hospital, and the citizens.

### THE HOLIDAY QUESTION AT LEYTON.

At a meeting of the Leyton Urban Council a report of a sub-committee recommending that the annual holidays of the matron of the isolation hospital be increased to a month, and that of the charge nurses and assistant nurses to three weeks, gave rise to an animated discussion. Several members of the Council objected to the proposal because the question was settled in December 1897; but as one member who was not on the Council in 1897 observed, there is no reason why the settlement of the Council should be like the law of the Medes and Persians. However, the opponents of the extension of the holiday were successful, an amendment to the recommendation of the sub-committee being carried by 13 to 9 votes.

### SHORT ITEMS.

We are informed by cable that Miss Helena Relph, matron of the Bangkok Nursing Home, which is under the Colonial Nursing Association, was married on July 20th to Mr. D. O. Witt, of the Indian Forest Service, at the Anglican Church, Bangkok, Siam.—An action by eight nurses of the Londonderry Workhouse Hospital against a female guardian for defamation of character has resulted in a verdict in their favour, with damages one shilling in each case for each plaintiff.

### The Nursing Outlook.

"From magnanimity, all fear above;

From nobler recompense, above applause,

Which owes to man's short outlook all its charm."

### THE SCHOOL NURSE ESTABLISHED.

After six short years the school nurse is established in London as a municipal officer; and in a brief time the Voluntary School Nurses' Society, which showed the way, will cease to exist. This is a very important and instructive fact, for it shows how valuable private charity may be in trying an experiment and in paving the path of proper public expenditure.

Last week, before the London County Council, the Education Committee reported that it had been the practice of the late School Board to employ nurses to visit the schools for the purpose of detecting ringworm and securing personal cleanliness among school children. The nurses were only appointed temporarily, to the number of four, and their time was nearly up. Also the School Nurses' Society, who employed five nurses for a similar purpose, had reported that they found difficulty in raising funds; and, says the report; "The work which has been performed by the society is of acknowledged importance to the community, and was much appreciated by the late Board." The medical officer desired to have in all a staff of 12 nurses working under him to visit the elementary schools, and this was granted last week without any hesitation. It was also agreed to raise the salary of the nurses; in future they are to commence at £80 and rise to £90 by annual increments of £2 10s. Be it remembered that the London School Nurse has her Saturdays and Sundays free, and regular holidays, so that it is delightful work for those who can sympathise with our little gutter-snipes, and who wish to have some time to themselves. Here, then, is the resolution passed by the London County Council:—"That authority be given for the appointment of six more nurses in the Public Health Department; that their salaries be in accordance with the scale, and that their duties be defined by and carried out under the direction of the medical officer."

Three days after this was passed there was a meeting of the London School Nurses' Society, in order that its swan-song might be heard. The resolution of the Executive Committee was to the effect that since the London County Council had decided to employ a staff of 12 nurses, the subscribers should empower the executive to wind up the affairs of the L.S.N.S., and divide any balance between the associations which had helped in the work. This was not received without discussion, there being some feeling that there was still room for voluntary nurses working side by side with the official nurses; but it was pointed out that the object of the Society from the very beginning had been chiefly to educate the School Board in its duty towards the bodily development of the children, and that it had never been contemplated to run a permanent society. The first nurses had proved, what had been denied, that it was possible to do away with dirty heads and sore eyes in the schools without coming into conflict with the parents or teachers, and to the very great benefit of the children; and it seems worth while to record that the first school nurse was Miss Rose Petty, now inspector of boarded-out children, whose tact and love of the little people showed how useful and how beloved a school nurse could be. The society has quietly accomplished its object. It has always had the support of The Hospital. Its presidents have been—first, Lady Aberdeen, and, secondly, Lady Windsor; and its honorary secretaries—first, Miss Honnor Morten, and, secondly, Miss Susan Lawrence. But the main supporter of the scheme was the chairman of committee, Lord Stanley of Alderley; and the man who has helped the voluntary nurse to become a municipal officer is Dr. James Kerr. So much for the history of a worthy and successful society. But its influence has extended far beyond London.

The hon. secretaries have had frequent requests for the rules and regulations of the school nurses, not only from England and Scotland, but also from the United States and the Colonies, and the example of London in sending visiting nurses to their poorer elementary schools has been followed far and wide. New York, which started its school nurses two years after London, has already enlarged its staff to 45, has founded a special hospital for the eye cases, and is altogether going ahead with its usual vigour—and its usual friction! For the nurses have come into conflict with other officials, and, after all, London's tortoise pace may perhaps win this hygienic race. In our country towns the Queen's nurses have been doing the work for long; Miss Amy Hughes, the inspector for country districts, has always been an upholder of school nurses, and did the actual work herself in Clare Market even before the formation of any society, and merely at the instigation of the school managers. It is to be hoped that before long every large town will have one or more school nurses; it is far more instructive to the smaller children than theoretical teaching of hygiene in class. To watch the nurse bathe the eyes with a separate swab on each occasion; to see her turn up her sleeves and wash her hands and forearms and scrub her nails between cases, and to learn from her how the dirt has poisoned and inflamed the broken chilblain, are practical lessons that are never forgotten. We know of no more useful way of fighting physical degeneration than by the employment of school nurses.

### Lectures Upon the Nursing of Mental Diseases.

By Robert Jones, M.D.Lond., B.S., F.R.C.S.Eng., M.R.C.P.Lond., Resident Physician and Superintendent of the London County Asylum, Claybury.

### LECTURE XVI.

In the last lecture we described somewhat fully the "water treatment" in regard to the insane. It is necessary to lay down very definite regulations in regard to the ordinary bath which every patient receives upon admission into asylums—public asylums at any rate—and which he or she continues to receive every week, oftener if required, during the period of detention, unless there are medical or other special reasons to the contrary. Should there be the slightest doubt as to the advisability of bathing any patient, owing to sickness, feebleness, or excitement, reference is to be made by the nurse, directly or indirectly, to one of the medical officers. The name of every patient not having the customary bath is to be inserted by the responsible nurse in her daily report of the ward. The charge attendant or sister of the ward is always to be present during bathing and the Lunacy Commissioners insist that an officer such as the matron, or someone deputed by her should also be present. We propose to relate, somewhat fully, the rules which are generally insisted upon in all asylums with regard to bathing, for very serious accidents—amounting even to fatal scalding—have taken place during bathing. It is customary in asylums to have the bath taps locked with a special key and clever devices have been invented whereby there is no possibility of turning the hot water for a bath without first turning on the cold so that the hot water first flows in this way through the cold. The bath key is used by the nurse alone, it should be attached with the other keys to the nurse's belt and should never be given to or employed by a patient, and during the employment of the bath the room is never to be left without a nurse or attendant, as some depressed patient may attempt to end life by drowning. When the bath-room is not used, the door is to remain locked, the taps secure, the floor to be kept dry and everything nice, clean, and orderly.

In preparing a bath—where the patent tap is not used—the cold water is always to be turned on first, but no cold bath is ever to be employed except under medical orders and then only for the duration of time specified and in the presence of one of the so-called officers, such as the matron or chief attendant. It is understood that the weekly or ordinary bath is used solely for the purpose of cleanliness, the body of each patient being well cleansed with soap. Before the patient enters the bath, the taps both for hot and cold water are to be turned off and locked, the water is to be well mixed, and the temperature is to be taken with the bath themometer, which should not register less than 90°, nor more than 98°. No additional hot or cold water is to be added to the bath whilst the patient is in it. In case the thermometer has become inaccurate from injury or other cause, all bathing operations are to be suspended until another instrument is obtained. While the patient is in the water, under no pretence is the head of the patient to be put under water, the hair is to be cleansed with soap, and water from the bath or from a can previously arranged douched over it to clear it from all soap. After the patient has come out of the bath especial care must be taken to dry those who are feeble and helpless and to clothe them as rapidly as possible. Not more than one patient is to be bathed in the same water, and under no circumstances whatever are two patients to occupy the bath at the same time. If there is any deficiency of warm water the bathing must be suspended, and the irregularity is to be at once reported and also entered in the daily report of the ward. Soap, towels, brushes and combs are always taken down by the charge nurse, or a special bath attendant, and the number of patients carefully counted before leaving the ward and bath-room and also after returning into the ward. Any marks, bruises, wounds, sores, local pain, evidences of disease of any kind complained of by the patients or noticed by the nurse during any of the bathing operations are to be immediately reported to one of the medical officers and also to be entered in the daily report of the ward by the sister or nurse in charge. The matron on the female side, and the chief attendant on the male side, enter also in their own reports that they have supervised the whole of the ordinary bathing operations and that the rules have been rigidly carried out, or, failing this, they name and describe the infringements thereof.

Reference has been made to the daily report of the ward. The following is a copy of the one in use at the London County Asylum at Claybury:—

Ward............... Date............... 190 .

**Evening Report.**

| | |
|---|---|
| Number of beds .. .. .. | Number of patients in bed .. |
| Patients in ward at bed-time .. | Patients in Airing Court, Morning Attendants do. .. |
| Vacancies .. .. .. .. | Patients in Airing Court, afternoon Attendants do. .. |
| New patients admitted .. .. | Patients Chapel, Morning .. |
| Patients received from other wards .. .. .. .. | Attendants do. .. .. .. |
| Patients sent to other wards .. | Patients Chapel, afternoon .. |
| „ discharged .. .. | Attendants do. .. .. .. |
| Absent on trial deceased.. | Number of patients beyond grounds .. .. .. .. |
| Temperature of ward .. .. | Number of patients in entertainment .. .. .. .. |
| Total number of fits .. .. | |
| Total number of cases wet .. | |
| „ „ „ dirty .. | |
| Total employed during day .. | |

Names of patients sleeping in padded rooms.
Names of wet cases.
Names of dirty cases.
Names of patients who have had fits or epileptic attacks.
Names of patients ordered to be in seclusion, and duration.
Names of patients ordered to be in restraint, and duration.
Accidents or casualties in the ward.
Names of attendants on duty.
Names of attendants absent.
Names of attendants employed outside the ward, and where.
Workmen or persons in the ward other than officers, and time.

**Patients Employed.**

| | |
|---|---|
| In laundry.. .. .. .. | At needle or fancy work in ward .. .. .. .. |
| dormitories .. .. .. | Cleaning corridors .. .. |
| workroom .. .. .. | Helpers in other parts .. .. |
| nurses' blocks .. .. .. | |
| the residences .. .. .. | |
| Helpers in the ward .. .. | Total employed .. .. |

**Patients Unemployed.**

Sick, infirm, and old age .. .. .. .. .. ..
Unemployed .. .. .. .. .. .. ..

Total unemployed .. .. .. ..

Names of patients outside grounds and attendants in charge.
Reports on other matters, patients visited, patients taking medicines, etc.

I have ascertained that all the patients in my ward are safe and all the doors securely locked.

(Signed)...................... Charge nurse or sister of the ward.

The day nurses in institutions for the insane generally enter upon their duties at six o'clock in the morning, and they remain on duty until 8 P.M. During this time they devote themselves exclusively to the care of the patients. They commence duty by unlocking the doors of the bedrooms or dormitories, and begin to hand their clothes to the patients, seeing that they dress themselves properly and with correct sequence. The nurse in charge of the ward visits the whole of the patients in that ward, as also the separate rooms—called "single rooms" because only one patient sleeps in each—with the night nurse, and signs a book that she receives them over correctly. The nurses assist those patients to dress whose mental condition or infirmity of body prevents them from doing so themselves, and they see that they are properly washed, that the state of the skin is free from soreness or discoloration, that their hair is combed, and that they are neat, clean, and tidy before breakfast. As soon as the patients have got up and are dressed, the bedrooms and passages are to be cleaned out, the beds and bedding laid out to air, and every kind of dirt, dirty linen, broken vessels, rags, remnants of food, and every description of litter removed and the windows opened. In summer and warm weather the windows and ventilators are opened shortly after six when the patients are sufficiently dressed, but in hot and dry weather instructions may be left for them to be open all night. Before seven o'clock the nurse has taken the foul linen—and this constitutes the daily in contrast to the usual weekly washing—to the laundry, and another nurse is sent to the kitchen or stores for the bread, butter, and other necessaries required for the day, but in some institutions these are drawn in the afternoon. Before eight o'clock in the morning all broken windows, furniture to be repaired, or other necessary repair is to be reported to the matron or her deputy, so that they may be entered in the daily report of repairs to the engineer or the clerk of works. From eight to half-past eight the patients' breakfast is served, care being taken that those who are in their rooms are also at the same time duly served. The strictest attention is to be observed in regard to the administering of morning medicines, baths, or other instructions directed by the medical officer. After breakfast—before breakfast in some institutions—the patients are collected and conducted to chapel. It is often most difficult for the nurse to select patients for chapel attendance, and although it is her duty to encourage their presence, those who are most desirous and wishful to attend are least fitted mentally for the consolation of spiritual ministrations. All the epileptics desire to be in chapel, and all cases of religious mania also desire to attend. These are often aggravated in their mental states by attendance, and it is best that the selection in doubtful cases should be decided by the medical officer. All patients attending chapel should be dressed in a neat, decorous, and becoming manner. Those who are liable to be seized with fits are seated near the door, as also those who may, under the influence of delusions, behave violently, so that they may be removed by the nurses with promptitude and quietness. Those whose conduct is the most doubtful should always be seated near the attendants. Those patients desiring to partake of the rites and sacraments of the Church are always in institutions referred by the nurses to the medical officer, and to the chaplain or spiritual advisers in attendance. It is a rule in all institutions that those patients desiring the ministration of their own denomination be accordingly given facilities for the purpose.

*(To be continued.)*

### New Nurses' Home at Gloucester.

### SIR MICHAEL HICKS-BEACH ON MODERN NURSING.

On Saturday Sir Michael Hicks-Beach, who was accompanied by Lady Lucy Hicks-Beach, formally opened the commodious nurses' home which has been erected in the grounds of the General Infirmary at Gloucester. The home comprises a central block and one wing, and has a basement for storage, and three floors, the great majority of the rooms opening upon the beautiful gardens with a southerly aspect. On the ground floor there are seven nurses' bedrooms, nurses' sitting-room, matron's sitting and bed rooms, and sanitary accommodation. There are also a small kitchen, larder, and a bicycle house on the ground floor. On each of the first and second floors there are 12 bedrooms for nurses, a couple of bathrooms, and other accommodation. The present provision is thus for a matron and 31 nurses, and due care has been taken for the quietude and seclusion of the sleeping quarters of the nurses when on night duty.

On account of rain the opening ceremony, which was to have taken the character of a garden party on the lawn in front of the Home, took place in the out-patients' hall, where the Bishop of Gloucester commenced proceedings by dedicating the Home to the honour and glory of Almighty God.

Sir Michael Hicks-Beach then delivered a brief speech. Having referred to the improvements effected in the infirmary itself, he turned to the more attractive and not less important side of that effort. The provision of a Nurses' Home marked, he did not say a new departure, but a very great step in what was a completely new departure in a very important part of healing work in this country. The Bishop would remember the time, which he himself could almost call to mind, when trained nurses were unknown. The idea of a nurse was something of this kind: any creature in female form—the older and the uglier the better, and the less suited for anything else the better too—was by a kind of poetic glamour presented by our predecessors as the ministering angel when pain and sickness wrung the brow. Well, they lived in days when, perhaps, poetic glamour had lost force. The change, begun by the great Miss Florence Nightingale, was at the time of the Crimean war, a revolution which would last, he hoped. For centuries Miss Nightingale's name would never be forgotten in connection with the nursing movement of this country. She taught people what might be done by trained nurses in war time. She taught that a good nurse required many, perhaps, he might say, all the best, qualities of a lady—gentleness, patience, deftness, method, sympathy; and through her work in the hospitals of the war there began a movement which soon extended to their great institutions of that kind in England, and which had now gone throughout our private and social life. He supposed that no skilled operator would care nowadays to be without the aid of a trained and experienced nurse at the time of an operation. With this great change in the nursing profession there had come a great need. The old-fashioned nurses to whom he had referred might be crowded together in unsanitary or small rooms; might be neglected, as they often were, without, he supposed, very much, comparatively, injury to themselves. But if they wanted a skilled instrument they must take care of it, even from the lowest point of view, in order to secure efficient work from the efficient nurses, whom, thank God, they had among them to-day. They must take care that they were properly lodged, lodged in healthy places, and that they had some means of quiet, simple comfort when they were off duty. Having appealed for funds in respect of the deficit on the expenditure, Sir Michael then opened the home, and a hearty vote of thanks was accorded to him and to Lady Lucy Hicks-Beach for their presence.

### Reception by the Queen of the Nurses of the Royal National Pension Fund.

BY OUR SPECIAL CORRESPONDENT.

THE sixth reception of the members of the Royal National Pension Fund for Nurses by the President took place on Friday, July 22nd. Intensely interesting as the event has always been to the nurses, it was rendered especially so this year because it was the first occasion on which the function has been held at Buckingham Palace; and from the time it was known that her Majesty the Queen had fixed the date of the ceremony, much pleasurable excitement prevailed among the members eligible to participate in it. Although heavy showers of rain fell less than 12 hours after the function, the weather was strictly in accordance with tradition, the glorious sunshine being tempered by refreshing breezes, and the heat by no means exhausting.

### INSIDE THE GROUNDS.

According to arrangement, the nurses who joined the Fund since the ceremony at Marlborough House in 1901 were admitted to the grounds of the Palace at the Constitution Hill entrance shortly before one. Owing to their work and other causes, a large number who are entitled to receive certificates are necessarily prevented from attending, and it was expected that the muster would be just under 1,000. This estimate was very near the mark, as 916 actually received certificates from her Majesty. In addition to these, the Queen had graciously consented to allow those nurses to be present who had previously been entitled to receive certificates but had been unable to attend; and the latter, to whom white cards were given, were admitted at two. About 300 took advantage of the privilege. There were a few late comers, but, generally speaking, the favoured guests were laudably punctual, notwithstanding the fact that many had travelled long distances that morning; and shortly after one the interior of the Palace gardens was thronged. A cloakroom on a considerable scale, equipped with all conveniences, had been thoughtfully provided, to which the nurses repaired as they arrived, and made haste to prepare for the preliminary drill which always precedes the reception. When the word was given to them to form into companies it was obeyed with alacrity, and in a few minutes the blocks of 100, into which they were divided, were complete. Each had a card sent her, showing the block to which she belonged—each block card being a different colour—and her number in that block.

### THE REHEARSAL.

The first part of the drill was gone through in close proximity to the cloak-room. Long before this, Mr. Louis H. M. Dick, the Secretary of the Pension Fund, and his capable staff, were on the spot, affording guidance and help to all comers, and before the proceedings commenced Sir Henry Burdett, the deputy chairman, nearly recovered from his serious accident, but still wearing his arm in a sling, arrived. Colonel Campbell, A.D.C., was the officer in chief command, and the three officers assisting him were Colonel Bor, Captain Cottingham, and Lieutenant Wright, all of the Royal Marine Artillery, the duty of marshalling the nurses being, as usual, undertaken by non-commissioned officers of the Royal Marine Artillery and the Royal Marine Light Infantry, who for the day were honoured with the title of "captain." The small percentage of nurses who carried parasols made use of them without exposing themselves to a charge of breach of military discipline. When the companies had fairly mastered the art of saluting they were marched to the beautiful stretch of lawn where the ceremony itself was to be held. Here, when they had been drawn up, Sir Henry Burdett went the round of the battalions, and delivered a brief address to each, expressing the hope that they would enjoy themselves. The rehearsal was not confined to the drill, in which the nurses marched four abreast and arm-in-arm to keep the line, but they subsequently, led by one of the "captains," filed before the royal daïs, so that they should understand precisely how to pass rapidly and in order. The quick response of A Company to the injunction to "stand up," "link arms," showed at once that the work of instruction had been efficiently performed.

### A GREAT REPRESENTATIVE GATHERING.

During the rehearsal it was possible to form a good idea of the representative character of the gathering. Every nurse, of course, wore Queen Alexandra's armlet, but this circlet of red was the only badge which was common to all. The uniforms were as varied as the faces of their owners. There were nurses with the scarlet of the Army Service, with the blue of the Navy, nurses in the uniform of the well-known London general hospitals, Queen's nurses in their bonnets with their badge and brassard, nurses in every colour from white to scarlet, nurses of every age, from the early twenties to the fifties, nurses of every height and of every type. Some were adorned with medals, others were decorated with the order of the Royal Red Cross. One of the most interesting of the recipients who wore this coveted distinction was Miss Bourguignon, matron of the Queen Victoria Memorial Hospital, Tientsin. In addition, she had the German Red Cross presented to her by the German Emperor and the Order of St. Elizabeth from the Emperor of Austria, all in recognition of her great and valuable services during the siege of Tientsin. A Ladysmith nurse donned the satche which was given her by the Queen; jewellery was conspicuous by its absence, and in many cases the perfect simplicity of the dress was a distinct gain to the wearer. Here and there was a face, or a figure, which suggested physical delicacy, but the great majority were healthy, comely, and graceful women, affording in their own persons a striking refutation of the theory that the occupation of nursing is detrimental to the body.

### THE SPECTATORS.

Hitherto there had been no band, but now the Irish Guards had arrived, and hundreds of eyes were turned in the direction of the Palace. But before the first note of "God Save the King" was struck, a few spectators who had been invited to witness the ceremony approached the red and gold canopy supported on silver pillars, presented to the King when, as Prince of Wales, he visited India, under which seats had been placed for the Royal party. They were chiefly the officers and committee of the Pension Fund and the Junius S. Morgan Fund, and those invited included the Duchess of Beaufort, the Marchioness of Zetland, Countess Cadogan, the Countess of Strafford, Lord and Lady Rothschild, Lord Aberdeen, Lord Aldenham, Sir William and Lady Broadbent, Sir Henry and Lady Burdett, Sir E. Cooper Perry, Mr. Everard A. Hambro, Mr. Eric Hambro, M.P., Mrs. Burns, Mr. Walter S. M. Burns, Mr. Thomas Bryant, Dr. S. H. and Mrs. Habershon, Dr. G. W. Potter, Mr. George King, Mr. R. Ernest Alexander, Mr. and Mrs. T. C. Dewey, Mr. and Mrs. J. Pierpont Morgan, Junr., Sir John Watney, Mr. E. Rawlings, Mr. and the Hon. Mrs. Charles W. Trotter, Mr. and Mrs. Falconer L. Wallace, Mr. Archibald Williamson, Miss S. A. Swift, Miss Katharine Monk, Miss Pauline

Peter, Miss Florence Smedley, Miss Mabel Cave, Miss Sophie Morris, Miss E. Vincent, Mr. and Mrs. Louis H. M. Dick, Mrs. Pritchard Binnie, and Mrs. Bretland Farmer.

### The Ceremony.

Next came in resplendent uniform the King's four Indian orderlies, under Captain McBarnett, D.S.O., and then, shortly after three o'clock, their Majesties entered the gardens from the door on the south side of the Palace. The King, wearing a grey frock-coat, the Queen in a costume of pearl-grey *crêpe de Chine*, with bolero, the Princess Victoria, Princess Mary of Wales and two of her brothers, and the Duke of Sparta, formed the Royal party, the suite in attendance including Colonel Brocklehurst, C.B., Major-General Sir Stanley Clarke, Colonel Davidson, Colonel the Hon. H. Legge, General Sir Dighton Probyn, Lord Farquhar, Lieut.-Colonel Frederick, Lord Kintore, the Hon. Charlotte Knollys, the Hon Violet and the Hon. Dorothy Vivian, Lady Gosford, and the Hon. Sidney Greville. Directly the Royal party reached the canopy Colonel Campbell gave the command—"Queen Alexandra's Nurses, Royal Salute," and the distribution of certificates began. It would not be easy to conceive a prettier sight than was presented during the march past. The background of stately timber, the lake on the right, the charming vista on the left, and the groups of nurses on the sloping lawn waiting to be presented, constituted a picture not likely to be forgotten by those who had the good fortune to behold it. The Queen, who stood the whole of the time in the centre of the red-carpeted space beneath the canopy, had on her left the Princess Victoria and her little grandchildren, who manifested the liveliest interest in the proceedings; and on her right Sir Henry Burdett and Mr. Dick, who handed the certificates to her. For each nurse to whom she handed the document her Majesty had a gracious bow and a kindly smile; and if the curtsey of the recipient sometimes seemed a little too perfunctory, it was always deeply respectful. Now and again Colonel Campbell, by gently moving his stick, checked those nurses who, in their ardent loyalty, wanted to be sure they did not keep her Majesty waiting a second; but from first to last, there was no fault to be found with anyone. When the certificates had all been duly presented, the ceremony was completed by the march past of the 300 nurses who had been prevented from attending the reception in 1901, and their salutations were also individually acknowledged by the Queen. So admirably had the nurses been coached, so adroitly did the commanding officer and his colleagues perform their labour of love the entire function only occupied just over an hour.

### An Unrehearsed Incident.

It was noticed that two fair-haired nurses, who had received certificates, were asked to remain behind the canopy, and there was much speculation as to the reason. Presently, as soon as the march-past was over, they were requested to move towards the daïs, and the Queen shook hands with both of them, conversing with them in Danish. When it was known that they were natives of Denmark, who are pursuing their training in England, the compliment paid them by her Majesty was understood and appreciated. Blushing and delighted, Nurses Müller and Lovgren rejoined their company, and then, the whole of the companies having responded with an enthusiasm that did credit to their lungs to Colonel Campbell's call for three cheers for the Queen, they were permitted to break their ranks.

### Presentations to their Majesties.

Although most of the nurses promptly availed themselves of the opportunity, a considerable proportion lingered near the canopy, while a number of presentations were made to their Majesties by Sir Henry Burdett, the most interesting to them no doubt being that of Mr. Dick, whom the King thanked for his many years' services to the Fund. There were no speeches, and following the presentations the King and Queen moved away to the strains of the National Anthem, conversing as they went with the visitors, including some of the nurses.

### Tea and Recreation.

The invitation to disperse was coupled with the intimation that tea was served in the large marquees near the lake, and thither the nurses trooped gladly enough. Though they had well maintained their bright looks, many of them must have felt fatigued, and in need of refreshment. Lavish provision had been made for their wants, and they were soon all enjoying tea and strawberries and cream, and other delicacies. The majority preferred to have it out of doors, and servants hastening to supply them with chairs, the space in front of the tents was soon covered with happy, laughing tea-drinkers. Rested and refreshed, some wandered about the gardens, amazed at their extent and delighted with the lovely flowers and shady corners; others, who delayed their explorations, had the good luck to obtain a share of the roses and carnations which, having been used to decorate the marquees, were distributed among them, and will doubtless be treasured as mementoes.

### The Departure.

Thus a pleasant hour and more was spent, and as the nurses walked and chatted in the grounds it was not difficult to learn what they thought of the reception. Two came from Paris; one from Brussels; six arrived from Rhyl at 3.30 A.M., and returned the same night. One who had travelled from Liverpool and had to return to resume the care of her patient early on Saturday morning, said that "she would not have missed such a treat on any consideration"; another, who had come from Yorkshire and had as a matter of necessity almost gone without food that day until she had her tea, described drawbacks of that kind as the merest trifles; and a third confessed that when she joined the Pension Fund she hardly ventured to hope that it would be her proud experience to receive her certificate from the Queen in the grounds of Buckingham Palace. The testimony of a fourth, though convincing, had in it a touch of pathos. She was among the multitude of nurses who were slowly retracing their steps, and as they did so furnishing for the photographer—had he been on the spot—a singularly effective subject. With a suspicion of tears in her voice she observed, "This is the worst of it; the most lovely day must come to an end." Then with a smile she added, "But we shall always have the memory of it."

### TRAVEL NOTES AND QUERIES.

By our Travel Correspondent.

Criccieth or Port Erin (A. B.). I do not know much of the first, have only passed through it, but it is rapidly becoming a fashionable place. There are three hotels. Try the "White Lion" for one night and look for rooms. Port Erin is very pleasant and much quieter than Douglas, but not nearly so well placed for excursions. Douglas is nearly in the centre of the island, hence its attractiveness. If you want the address there write to me. It is not necessary to go to an hotel at Port Erin for the night. Leave your things at the station and look for rooms, there are plenty. September is not so dear a month as August and your expenses need not be much.

### Nurses at Buckingham Palace.

BY A NURSE IN THE NORTH.

It is about two weeks ago since I received an invitation to attend a reception for nurses to be held by Queen Alexandra in the grounds of Buckingham Palace, the occasion being the presentation of certificates to nurses who had joined the fund during the last three years. Although some distance from London I was determined to be present, if possible, and I was fortunate enough to be nursing in a house where my patient and family were so interested in the matter that they did all in their power to let me get off quite comfortably.

I had to take a journey of six hours. I was driven to the little country station about 11 o'clock in the day on Thursday, stayed two nights with a married sister in town, and returned to my case on Saturday, whilst a nurse was doing duty for me.

Friday, July 22nd, 1904, was a glorious day and a gala festival indeed for us nurses. We arrived at the gates of the grounds, Constitution Hill end, about noon, though we were not admitted till 12.45. I had only been standing for five minutes when I came across a nurse whom I had met last autumn. She was the only one I knew out of the 1,400 who were in the grounds, and I soon discovered that I was not the only lonely one in the crowd.

There was a tent erected as a cloak-room, but many nurses had come in their caps and aprons, some in hansom-cabs, some in omnibuses, brakes, motor-cars, and carriages and pairs, many walking from Victoria Station.

I think there must have been several hundreds of institutions represented, though on the whole blue or grey was the prominent colour of the uniforms worn. I and other nurses remarked how nice and neat the majority of nurses appeared, which, from what I had heard, was an improvement upon some former occasions. We each possessed a card with a letter and number; there were 100 nurses with the same letter, so that we formed up in lines of four, one line behind the other.

When all had been marshalled into order by the officers of the Royal Marine Artillery, directed by the Colonel in command, we had to go through a preliminary drill. The Colonel ordered a "Royal salute," calling us "Queen Alexandra's nurses," raised his hand, and we all raised our right hands with palms facing outwards. When we did this on the approach of the Queen the "National Anthem" was played, just two or three bars, whilst we kept our hands up. Then when it finished we let our hands drop to our sides, and they made such a "bang" altogether against our starched aprons.

It was rather a trying time standing in the sun for so many hours, and some of us did not escape a headache; but it would have been worth while even had the wait been longer to be received by our Queen in so gracious a manner and to gain our certificate from her own hands.

We were marched in companies from our stations to the right-hand side of the Queen, and as we walked in fours we had to link our arms in one another's. We then advanced singly before the Queen, made a bow or curtsey, received our certificate, bowed, and passed on, and then formed up again into a company, and finally marched arm-in-arm into our first-appointed place.

It was such a pretty sight, and I think all went off well. I spoke to several of the nurses, and they all said that they had enjoyed it very much. One had come from Dover, another from Brighton, another from Tunbridge Wells, and so on.

The ceremony commenced at three o'clock and lasted only an hour. We were to have formed up into a semicircle when it was over and to have been addressed by the King, but this part of the ceremony was dispensed with.

The tea was very acceptable and the floral decorations were very pretty, and we were allowed to have the flowers off the table. I was too late to get any, and so I begged a spray of carnation from a nurse just to keep as a memento. The tea-table was loaded with sandwiches, cakes, and fruit of every kind, as well as iced strawberries, coffee, and ice-creams. As we got served most of us sat on the grass and rested and chatted.

Rather an amusing incident occurred while we were waiting to be drilled. It was very hot standing in the sun, and the chairs a few yards off looked so tempting that some of the nurses could not resist the temptation to fetch them. But the deed was soon discovered by the officiating gentlemen, and some one in authority ordered the sergeants to stop it at once.

I am sure the sergeants must have felt amused many a time, for drilling nurses must be really more difficult than ordering the men about. They were really very good and considerate in every way to us, allowing sunshades and also keeping them safe for us when we went before the Queen, though a few nurses actually forgot and carried them in their hands right up to her Majesty.

As I crossed London next day to return to my work, the conductor of my omnibus, noticing that I was a nurse, began to chat about the ceremony, and I found that he had seen a good deal of it from outside. So I allowed him a peep at the certificate given me by the Queen herself, which he thoroughly appreciated, and I am sure that I went up many steps higher in his esteem when he knew what I possessed.

We nurses all feel most grateful to our King and Queen for giving us such an opportunity of seeing them, as well as for the privilege of being in the beautiful grounds of the Palace. There were so many, too, who had worked hard to make everything go off so well, that I feel we really owe a debt of thanks to all. But I hope the secretary will tell them in the way he thinks best. I should much like to thank each one personally. But this I know is impossible.

### The Registration of Nurses.

On Thursday, July 21, Mr. Walsh gave evidence before the Select Committee of the House of Commons with reference to male nurses. He stated that while the demand for male nurses was great and increasing, there was a lack of uniformity in training. Training in the wards of general hospitals, while very desirable, was not encouraged. Registration would raise the standard of nursing, and would be a protection to the better class of nurses, as well as to the public. He desired for male nurses the same opportunities of education as those afforded to women. Examination by a Central Board should be confined to nursing pure and simple. He would have no objection to the registration of nursing homes.

Evidence of Miss Hobbes.

The next witness was Miss Annie Hobbes, Secretary of the Royal British Nurses' Association. She pronounced herself distinctly in favour of registration, because she considered the only way to achieve uniformity in training would be by the establishment of a Central Board, and because it would lessen the demand for untrained nurses. She saw a certain difficulty in the removal of unsatisfactory names from the register, but thought this would be obviated by powers conferred on the Central Board to remove anyone who proved to be in any way criminal, or in any way criminally unfit for her work, *i.e.* proved to be so in a court of law; or for systematic neglect of duty, repeatedly alleged by medical men and employers. A doctor and even a patient should be entitled to make privileged communications to the Central Board, provided the matter were sufficiently serious.

The Chairman said that the essential point was as to the possibility of ascertaining whether a nurse were good or bad, to which the witness replied that there should be a sufficient staff of clerks to undertake the necessary inquiries. Each nurse should apply annually, on payment of a small fee, and the production of at least two written and genuine testimonials as to character and physical and technical fitness, from a registered medical practitioner, or some person in authority competent to judge. This system should be continuous, revision of the register being made annually, and the duty of application being imposed on the nurse. In the case of a member of a large co-operation, a testimonial from that body should be sufficient. She submitted that an annual fee of 2s. 6d. from each registered nurse would meet the cost of inquiry, three-guineas being the outside fee for examination. If nurses realised the desirability of being on the Register, the expense would be no deterrent.

Replying to questions, Miss Hobbes said that many nursing homes were sending out partially-trained nurses for whose services full fees were charged, and that many of these nurses were incompetent. Registration would to a great extent eliminate the danger, by putting the public in a position to choose properly trained women; of course, however, some people would go their own way, whatever was done in their interests. She understood that cottage nurses would be untouched. There might be two classes, the partially trained being left as at present; neither asked to pass any examination nor to pay a smaller fee, this being a course which she thought would lead to difficulties. Registration would be carried out under various sections, each stating to what class the nurse belonged. For those who from any reason dropped out of work, re-entering the ranks later, there should be some system of post-graduate instruction, so that, being re-taught and re-examined, these women might have their registration renewed. Testimonials would, of course, be required.

Dr. Hutchinson suggested that there would be a difficulty in insisting upon every nurse being given the training and experience desired by the witness, to which Miss Hobbes replied that what was wanted was the opportunity; some nurses under present conditions being rather made use of than taught. Even for 85,000 nurses, she thought that there was room enough in the hospitals. The opponents of registration laid great stress on character. This was of undoubted necessity, but if it were all important, why, she asked, go through a long and sometimes tedious training? She saw no reason why registration should prevent inquiry; this was generally made with respect to character, the proposed scheme being designed to ensure proper qualifications to do the work required. She believed that registration would create a more earnest class of nurse, besides raising the status. She was not apprehensive that it would diminish the number of nurses.

The Chairman having asked for observations on the manifesto, which was signed by 484 nurses, in addition to persons in authority, Miss Hobbes thought that it would have been more important to have obtained the signatures of nurses no longer connected with hospitals. It was natural that those in hospital should act from a sense of loyalty towards the institution with which they were connected; a nurse, moreover, rarely began to think independently until free of the hospital to which she had been attached. Sir John Talbot asked whether the same remark would apply to members of the Royal British Nurses' Association, to which the witness replied that nurses knew the objects of the association before joining, and that membership was entirely voluntary. The chairman having asked whether the witness suggested that those who signed the manifesto, while acting for the best within their lights, were not very conversant with the conditions, Miss Hobbes gave an affirmative reply.

In reply to other questions, Miss Hobbes said that about 120 names were annually added to the register of the Royal British Nurses' Association after most exhaustive inquiries. She concluded that the State would accept the certificate of the Association in the first instance, and that a representative would sit on the Central Board.

In reply to Dr. Hutchinson, Miss Hobbes said that the registration of nursing homes would be a step in the right direction, though not a sufficient one. The object of registration was to prevent anyone from nursing without proper training and calling herself a nurse.

### Evidence of Miss Amy Hughes.

Miss Amy Hughes, superintendent of the County Nursing Associations affiliated to the Queen Victoria Jubilee Nursing Institute, gave evidence on Tuesday.

Having stated the case for registration, Miss Hughes suggested that legislation for nurses should be on the lines of the Midwives Act. The register should be periodically revised —say, every three or five years, as found convenient by the Central Board, the nurses notifying themselves for the purpose. She considered that Parliament had already faced the question of the elimination of undesirable names in the Act referred to, and that there would be no difficulty in following the same plan with regard to nurses. Removal on such grounds as carelessness or impatience would, she said in reply to a question, have the effect of eliminating all but saints. A voluntary sub-committee might be formed from the Central Board (the board to be composed of matrons, medical men, and an equal number of lay persons interested in the subject) to investigate quietly, and, if necessary, caution the nurse in question without proceeding to the stringent measure of removal. She placed the total number of nurses likely to register as much nearer 20,000 than 80,000. The nursing register would be voluntary, and would simply protect those women who had given time and energy to the extra training required.

Being asked whether those nurses engaged in nursing the sick poor would be likely to register in large numbers, Miss Hughes thought not. The majority of the village nurses married after a short period of service; a small proportion, however, might wish to continue their training later in their career, and to qualify for registration. She saw no difficulty in the reinstatement of a nurse who desired to re-enter the ranks after discontinuing her work, through marriage or other causes. The necessary inquiries could be undertaken by the sub-committee. Registration, in her opinion, would increase the demand for fully-trained nurses, and the levelling-up of all the hospitals would attract larger numbers of women. Two or three guineas was not too high a fee for examination, and she thought it would be paid willingly. Uniformity would never be obtained, but by means of examination weak points would be discovered, and alterations might be made; registration would help to show that every woman had received full instruction in all details of practical work. Poor-law infirmaries must be considered on account of the responsible nature of their work. She thought there would be plenty of room in the hospitals for the number of women wishing to train if the smaller ones were levelled up—the large ones had applications far in excess of vacancies.

In reply to Dr. Ambrose, Miss Hughes said there were 220 village nurses in the association she represented; the system of inspection in the homes of the poor was not found to give rise to any inconvenience. The same member seemed to think it a hardship to expect a nurse, in addition to her three years' training and the gaining of a hospital certificate, to further undergo a State examination, with the payment of a fee.

In reply to Dr. Hutchinson, the witness said the proportion of bad nurses—*i.e.* those who brought discredit on their profession—judging by her own experience, was small. The registration of nursing homes would only shift the responsibility, which, she maintained, should be borne by the individual. The suggestion was an admission that something needed to be done, but it did not sufficiently meet the case. State registration should not touch moral qualities, but [should ensure a much higher standard of technical skill, and a greater degree of uniformity in training. A State examination should emphatically include *viva voce* questions.

### The Central Midwives Board.

A Meeting of the Central Midwives Board was held at the office, 6 Suffolk Street, on Thursday last week. There were present the Chairman (Dr. Champneys), Mr. Ward Cousins, Miss Oldham, Miss R. Paget, Miss Wilson, and Mr. Parker Young.

A letter from the Registrar-General in reply to a communition with regard to the death certification of midwives was read. The question under discussion being the need for calling the attention of local supervising authorities to the duties with which they are charged under the Act of notifying to the Board the deaths of midwives within their areas, it was agreed that the County Councils Association should be referred to, and a letter written to Mr. Heywood Johnstone, the representative of the Association on the Board.

A letter from the town clerk of Brighton was next dealt with, including correspondence with regard to a midwife, who had caused the death of a patient, and had disobeyed the injunctions of the medical officer of health by going to another case from one of scarlet fever. The Board agreed that it was the duty of the local authority to have suspended the woman and have communicated the facts to the Board. The secretary was instructed to quote at length the clause of the Act dealing with such cases, and to ask for an official report.

Several institutions, whose applications for recognition had not been accepted, having written asking for an explanation of their rejection, it was agreed that a similar reply should be sent in each case, referring the applicants to the requirements of the Board, and to their own inability to fulfil these, as shown by the answers in the schedules; adding that as soon as any institution might be able to comply with the requirements the Board would be most happy to reconsider their application.

Other letters were considered, including one from the Secretary of the Obstetrical Society of London, giving information with regard to certain irregularities in signing certificates from a lying-in charity. The applications of 539 midwives for certificates were approved, and ordered for entry on the roll, bringing the total number enrolled to 6,698. The Newport and Monmouthshire Hospital was approved as an institution for the training of midwives under Section C. of the rules.

A long discussion upon a resolution of Mr. Ward Cousins to the effect "that registered members of the medical profession only shall be eligible for appointment as inspectors under the Central Midwives Board" resulted in its withdrawal, no other member endorsing Mr. Cousins' views. Another resolution, also in Mr. Cousins' name, relating to the examination of candidates, was deferred. It was moved from the chair, and agreed, that the sanction of the Privy Council be asked to an alteration in Rule C.I. (2), the substitution of the words "under supervision satisfactory to the Central Midwives' Board," for "to the satisfaction of the person certifying."

The date of the next meeting was fixed for September 29th.

### Everybody's Opinion.

AN EXPERIENCE IN A PRIVATE NURSING HOME.

"Justice" writes: I shall be glad if you will kindly publish the following details, being my experience in a private nursing home not 100 miles from London, on the staff of which I was employed for over two years. I was sent to the town's isolation hospital to nurse a special case of fever—night duty only. My patient so far got well in about a week, and the ward I was nursing in began to be filled up with fresh arrivals, who were also placed under my charge. I was next requested to nurse the patients in the daytime, and also sleep near them, in order to attend to their wants at night. This I refused to do. The patients were not convalescent, some having just arrived, and with high temperatures. Our printed Institute rules were to the effect that "seven hours complete rest out of the sick-room" was to be granted to the nurses at their cases. This I quoted to the superintendent, but was simply told I could not remain on the staff if I refused to do as I was asked, and accordingly I sent in my resignation. No quarantine fee was allowed me, neither was I permitted to return to the institute till I had been in quarantine, so I had to go away for a week at my own expense. I hope such injustice is not practised in many institutes, but I rather fear a good many of these places want showing up.

### Appointments.

Bideford Infirmary.—Miss R. Budd has been appointed staff nurse. She was trained at Portsmouth Workhouse Infirmary.

Cardiff Union.—Miss Christina Perrin has been appointed charge nurse. She was trained in the City of London Infirmary, where she has been staff and charge nurse. She has also done private nursing in Southsea.

Eltham Cottage Hospital.—Miss Elizabeth Linton has been appointed staff nurse. She was trained at Burton-on-Trent Infirmary, and has since been charge nurse at Birmingham Ear and Throat Hospital, and temporary nurse at Bridgwater Infirmary. She has also been on the private nursing staff of the Royal Berkshire Hospital, and nurse-matron at Liskeard Cottage Hospital.

Hackney Union.—Miss Beatrice Garbutt has been appointed superintendent night nurse. She was trained at the Walsall and District Hospital, and has since been on the staff at Lowestoft Hospital, Ancoats Hospital, Manchester, and the Royal Hospital, Bristol.

Leavesden Asylum, King's Langley.—Miss Grace Mary Stewart has been appointed superintendent nurse. She was trained at Ashton-under-Lyne District Infirmary, where she has since been sister. She has also been nurse at Inverness Asylum, and night superintendent and day sister at Kent and Canterbury Asylum.

Northampton General Hospital.—Miss Emily Homesfield has been appointed sister. She was trained at the Royal Infirmary, Derby, and has since been sister at the Children's Hospital, Birkenhead.

Royal Ear Hospital, London.—Miss Annie Phillips has been appointed matron. She was trained at King's College Hospital, and has since been sister at the National Orthopædic Hospital, London; sister at the Victoria Hospital for Children, London, and matron of Tiverton Infirmary.

Royal Halifax Infirmary.—Miss Ethel M. Swinton has been appointed sister of the male surgical ward, and Miss Jessie Milroy, sister of the children's ward. Miss Swinton was trained at Walsall and District Hospital. Miss Milroy was trained at the Royal Halifax Infirmary, and has also been nurse at Walton Union Infirmary, Liverpool.

### Echoes from the Outside World.

### The Queen and the Salvation Army.

The Queen received General Booth at Buckingham Palace on Saturday. When the head of the Salvation Army visited the King just before the opening of the International Congress the Queen was not at the Palace. On Friday he received an intimation that her Majesty desired to see him on the following day. The Queen, in the course of the interview, assured General Booth of the sympathy which she has long felt for the great body of devoted officers which he has gathered around him in various parts of the world.

### Royalty and Horticulture.

The King, accompanied by the Queen and Princess Victoria, opened on Friday last the new hall and offices of the Royal Horticultural Society, which have been erected in Vincent Square, Westminster, to celebrate the centenary of the society. They arrived about half-past twelve and, considering that the day was exceedingly warm, must have been agreeably surprised at the comparative coolness of the white stone hall, where the ventilation was excellent and the sun's rays cleverly tempered by a shaded roof. The arrangement of the hall was very picturesque. The platform had quite a regal appearance with its crimson baize and Turkey carpet, the gilt chairs in the centre standing out against a bank of palms, Japanese maples, white hydrangeas, and lilies. On the wide tiers of steps many smaller chairs, equally gilded, had been placed, and the decorations were confined almost entirely to red and blue. Sir Trevor Lawrence read an address stating that the society was formed in 1804, and that to celebrate its centenary the present hall had been built, whilst, owing to the generosity of Sir Thomas Hanbury, a celebrated garden had likewise been acquired in the place of the one at Chiswick, which had become unsuitable for the purposes of the society owing to the advance of suburban London westward. He also mentioned that the number of Fellows had risen from 1,300 in 1887 to 8,150 at the present time. The King, in returning thanks for the loyal and dutiful address, hoped that the opening might prove the occasion of an accession of prosperity to the Royal Horticultural Society. Before leaving the Queen accepted a beautiful bouquet of orchids, and Princess Victoria one of Malmaison carnations, from Lady and Miss Lawrence respectively.

### The Prince of Wales and Sea Service.

On Friday the Prince and Princess of Wales visited the *Worcester*, the Thames nautical training college, in order to present the prizes to the cadets. The presence of the Princess was especially noteworthy, it being the first time that a Princess of the Royal House has visited the ship. Her Royal Highness handed the King's Gold Medal to Mr. Harold Bishop Stylehurst as the cadet who showed the qualities likely to make the finest sailor. The Prince of Wales, speaking as a sailor with 20 years' experience, and as one who had gone practically through the same training as the *Worcester* cadets, asked the cadets to keep before them two guiding principles—to be loyal to King, country, and ship, and to "be thorough." Let them put heart and soul into their work. To his mind, sea service was the finest service that any man could adopt.

### The War in the Far East.

The seizure by a Russian warship of the *Malacca*, a ship belonging to the Peninsular and Oriental Steamship Company, was followed by a protest to the Russian Government by the British Ambassador at St. Petersburg on behalf of the British Government. It has since been announced that the Vladivostock Squadron sank the British steamer *Knight Commander*, from New York, off Idzu on Sunday, after transferring the crew to another British steamer, the *Tsinan*, which arrived at Yokohama on Monday; and have seized the P. and O. steamer *Formosa*. The Prime Minister on Monday told the House of Commons that the great difficulties arising out of questions of this kind were causing the Government great anxiety, but the signs portended a favourable issue. With regard to the war itself, General Kuroki, in a report from Tokio, gives particulars of the recent fighting in the neighbourhood of Si-ho-yen. The Japanese casualties were 72 killed and 452 wounded. The Russians left 131 corpses on the field, and their casualties are estimated at over 1,000. Japanese troops have entered Niu-chwang.

### Storms and Disasters.

On Monday London and the country generally were visited by thunderstorms of considerable violence. In the metropolis the temperature, which had risen to 73° during the day, fell to 63° before eight o'clock in the evening. During the storm, which began to rage between six and seven, a train on the Metropolitan Railway ran into a flood in the tunnel between the stations, and the water rose to the seats of the carriages, the passengers being detained in a very trying situation for over an hour. There were several fatalities from lightning A young man was struck dead as he was walking along Stamford Street, Blackfriars, and fell through a provision shop window. At Barmouth, a well-known football player, who was strolling with his wife on a hill overlooking the town, was killed, and his wife was seriously injured. A farmer who was ploughing with two horses at Holtfleet, near Worcester, was also killed; and in Yorkshire a young man had his side paralysed. Fires and floods are reported from several localities.

### The Late Mr. Wilson Barrett.

Last week Mr. Wilson Barrett died of failure of the heart's action after a successful operation for cancer in a nursing home in London. He was 58 years of age. As a boy—he was the son of an Essex farmer—he was never allowed to enter a theatre, but he was so fond of plays that before he was 11 he had committed to memory the whole of "Hamlet" and "Othello." At his first engagement at Halifax he seized an early opportunity to take the part of an actor who was incapacitated, and soon made his way upwards. In 1877 he played in "Jane Shore," the title part being undertaken by his wife, Miss Heath, who had been reader to Queen Victoria. Later on he took the Court Theatre and played with Mme. Modjeska in many adaptations from the French, but his greater successes came later when he produced G. R. Sims' drama "The Lights of London" at the Princess's. This was followed by "The Silver King," in which Mr. Barrett played for over 300 nights without a break, and also performed a similar feat in W. G. Wills' poetic drama "Claudian." Mr. Barrett played "Hamlet" with considerable success, but it was in the famous "Sign of the Cross"—written by himself—that he achieved his most signal triumph.

### The August Bank Holiday.

The new service inaugurated by one of the New Palace steamers—the *Koh-i-Noor*—to Deal and Dover and back in a day seems to be greatly appreciated, and will doubtless be largely patronised on the August Bank Holiday. The facilities offered by this route include special trains, which run in connection with the sailings of the *Koh-i-Noor*, and call at important stations between the London terminus and Tilbury. The company have lately made an alteration in their tariff for refreshments in the second-class saloon of the steamers, and an excellent meal is served for a most modest sum.

### Notes and Queries.

**FOR REGULATIONS SEE PAGE 143.**

*A Question of Claim.*

(129) I am a trained nurse. I went to a heavy case 70 miles from London, after a few days I met another nurse coming at the door and found I was dismi-sed, as I was not considered cheerful enough. Can I claim fares to and from London?—*Manhattan.*

An arrangement should have been made before going to the case. The question being a legal one, you had better consult a solicitor.

*Home.*

(130) Can you tell me of a home which, for a small fee, would take a helpless young man of the middle-class?—*Nurse W.*

See Homes under "Chronic and Incurable" in "Burdett's Hospitals and Charities," or advertise.

Will you kindly tell me of a convalescent home on the east coast (not Kent), where a nurse who is unable to walk, but otherwise in fair health, could be received for about £1 1s. a week?—*J. M.*

See "Burdett's Hospitals and Charities."

Will you kindly tell me where a lad of 18, completely paralysed, could be received for payment during a few weeks, pending other arrangements?—*A. B.*

Advertise your requirements.

Can you tell me of a home where a respectable young woman of 30, suffering from caries of the spine, could be received free, or for a very small payment?—*Assistant Superior.*

See reply to *Nurse W.*

*Hospital Training.*

(131) (1) Will you kindly tell me if Bradford Royal Infirmary is attached to a workhouse or not; (2) if it is a recognised training school for nurses?—*An Inquirer.*

(1) No. (2) It is a large training school for nurses.

Will you kindly tell me of a hospital or infirmary where probationers of 20—if that is not too young—are taken?—*C. M.*

See "The Nursing Profession: How and Where to Train" for list of hospitals and terms for training probationers. General hospitals will not receive you till twenty-three.

Will you please tell me if I could get into a general hospital as probationer at my age, 33?—*M. J.*

Yes. See reply to *C. M.*

Can you kindly tell me of any nursing home where young girls can get training free?—*A. B.*

See reply to *C. M.*

*Sea-going Nurses.*

(132) Will you kindly tell me of any line of steamships which take trained nurses as part of the crew, and say which is the best way to apply for an appointment?—*F. E. B.*

Write to Miss Penn, The Cottage, New Shoreham, Sussex, but we are afraid that this movement has fallen through.

Can you kindly inform me how to become a stewardess? Also, what experience is necessary, the age, pay, etc.?—*Nurse E. S.*

See reply to *F. E. B.* and write to the secretaries of the various liners.

*Maternity Training.*

(133) Will you kindly state how many cases it is necessary for a monthly nurse to see during training in order to be proficient; and should she be taught to examine patients?—*A Three-Years' Nurse* and *Nurse H.*

Twenty cases is the minimum for midwifery training, and we hold that a monthly nurse should not have less practical experience, and that she should learn how to examine patients.

Will you kindly let me know of an institution where training is given free in monthly nursing?—*A. S. W.*

There is no free training. See reply to "*A Three-Years' Nurse.*"

### Handbooks for Nurses.

"How to Become a Nurse: The Nursing Profession." 2s. net; 2s. 4d. post free.

"The Nurses' Dictionary." By Honnor Morten. 2s. post free.

"A Handbook for Nurses." By J. K. Watson, M.D. 5s. net; 5s. 4d. post free.

"A Practical Guide to Surgical Bandaging and Dressings." By W. Johnson Smith, F.R.C.S. 2s. post free.

"The Art of Massage." By A. Creighton Hale. 6s. post free.

"Preparation for Operation in Private Houses." By Stanmore Bishop, F.R.C.S. 6d. post free.

The above works are obtainable of all booksellers, or direct from The Scientific Press, Limited, 28 & 29 Southampton Street, Strand, London, W.C.

### For Reading to the Sick.

### THE CROWN OF LIFE.

AND if the trouble should be hard to bear,
And if the sorrow should be past compare,
And all the glory of thy life seem fled—
Know that the Father sorrows in thy sorrow;
That clouds to-day may mean a bright to-morrow;
That joy and gladness never can be dead.

For sorrow cometh but to bring thy soul
Out of the mazes of the world's control
To where the lamps of heaven are nightly set:
From out the pain there cometh blessèd peace—
And all the discord of the day shall cease
Upon the slopes of restful Olivet.

Thus shall thy weakness turnèd be to strength,
Thy tired heart find pleasant paths at length—
No more by restless fears and doubts cast down.
In all thy griefs God's tenderness to trace;
To know the sweetness of Love's pitying face—
Even this shall prove for thee Life's triumph-crown.

*W. G. Kingsland.*

And it has been pointed out that joy was the dominant note which rang through the first proclamations of Christianity. "Sorrowful, yet always rejoicing," is the very watchword of the Christian. It is Joy which is in the very front of our Saviour's teaching in the Beatitudes; it is His last legacy before His Passion, "These things have I spoken unto you, that My joy might remain in you, and that your joy might be full." "Your sorrow shall be turned into joy." "Your joy no man taketh from you." "Ask, and ye shall receive, that your joy may be full."

The doing His will is to tread in those paths where we most certainly shall meet Him and be cheered by Him. His ways are ways of pleasantness, and all His paths are peace. "He maketh me to lie down in green pastures; He leadeth me beside the still waters." Yes, when God asks us to promise, He is making us feel that we are dealing with a personal God, not with a mere abstraction, or an automatic punisher of right and wrong, but with a God who prevents us in all our doings, and who furthers us with His continued help, who helps us to end in Him with subsequent as well as prevenient grace. The road of promise which we traverse is flooded with the light of heaven, built up on the never-failing word of a bountiful God. "By myself have I sworn, saith the Lord." The river of grace flows beside it, and angels bear us up, and saints beckon us on; ending in that land which once more is "a land of promise," the land which He swore to our forefathers that He would give unto us. Grace is given me to live well; when I need it there will be grace given me to die well. "For Thou art with me." Now is the time to make firm that companionship. To be still, and know that He is God. To find the guiding hand in all its strength and security amid the death and life of each day's hopes and fears. And when we enter the shadow, still it will be, "with God onwards."—*Canon Newbolt.*

Nor hath thy knowledge of adversity
Robbed thee of any faith in happiness,
But rather cleared thine inner eyes to see
How many simple ways there are to bless.

